# Weak inter­actions in crystals: old concepts, new developments

**DOI:** 10.1107/S2056989018005339

**Published:** 2018-04-17

**Authors:** Andrei S. Batsanov

**Affiliations:** a Department of Chemistry, Durham University, Science Site, South Road, Durham DH1 3LE, UK

**Keywords:** crystallography, weak interactions, editorial

## Abstract

Andrei Batsanov presents a general review of weak interactions in crystals, which highlights the history, state-of-the art and future developments of this field.

## Introduction   

In structural chemistry and crystallography, the term ‘weak inter­actions’ usually brackets together everything weaker than a single covalent bond or an electrostatic inter­action between directly contacting fully charged ions of opposite sign (*i.e.* an ionic bond). Because it is these forces that hold together a mol­ecular crystal, their study is almost synonymous with the science of organic crystal chemistry – and would require volumes to review. The purpose of the present paper is much more modest: to draw attention to the recent fascinating developments in this field (while also briefly tracing their historical roots) and some unfinished business of the past which now can, and should, be reassessed – and, of course, to provide an introduction to the following research papers.

The theme of this issue is an *integrated* approach to weak inter­actions in crystals. The all-too-common pitfall in a crystallographic paper is to make the discussion of geom­etrical details an end in itself. However, what makes crystal (and mol­ecular) structures stable and drives chemical reactions and phase transitions, is not the geometry *per se* but free energy, which is not so easy to visualize. Both, in turn, must be stepping stones to understanding, predicting and (hopefully) engineering the properties of crystals. All along, the crystal needs to be seen in a dynamic, rather than static, way – from thermal vibrations to phase transformations to solid-state reactions.^1^

## The scope: types of inter­actions   

Physically, these weak inter­actions can be classified into Coulombic forces between (usually) not very polar species, the effects of mutual polarization between mol­ecules (polarization forces), the dispersion (van der Waals) forces, and the forces of mutual repulsion between closed electron shells due to the Pauli exclusion principle. The latter, of course, are ‘weak’ only at or near the equilibrium inter­molecular distances and increase exponentially when mol­ecules are forced closer together under pressure, quickly becoming anything but weak. All these forces are ubiquitous in all mol­ecular crystals – and beyond, in amorphous solids, liquids and even gases.

Several chemically specific types of weak inter­actions are often singled out. The hydrogen bond is by far the most important and the most studied – in fact, this concept even predates (Moore & Winmill, 1912 ▸) the discovery of X-ray diffraction. Originally, this term was applied only to *D*—H⋯*A* inter­actions where both donor (*D*) and acceptor (*A*) were very electronegative atoms (O and N, but also F and Cl). The crucial role of such bonds in the structure of water (ice), proteins and DNA is well known. These bonds have energies of *ca* 20–40 kJ mol^−1^, while the strongest (charge-assisted or resonance-assisted) hydrogen bonds of *ca* 150 kJ mol^−1^ are comparable in energy with covalent bonds proper. Later the concept of the ‘hydrogen bond’ was expanded, with substantial controversy (Bernstein, 2013 ▸), to include ‘weak’ hydrogen bonds, *e.g*. C—H⋯O (*ca* 5 kJ mol^−1^) and C—H⋯π, which can have energies as low as 0.2 kJ mol^−1^, imperceptibly merging into ‘unspecified’ van der Waals inter­actions. More recently, Metrangolo and co-workers (Metrangolo & Resnati, 2001 ▸; Metrangolo *et al.*, 2005 ▸, 2006 ▸) introduced the concept of the ‘halogen bond’. Mono-coordinate Cl, Br or I atoms (*X*) have a depletion of electron density (σ-hole) opposite to the covalent bond (Politzer *et al.*, 2007 ▸), therefore *Y*—*X*⋯*D* contacts with electron-donor atoms *D* can be stabilizing with the energy varying widely, from 10 to 200 kJ mol^−1^. In a similar vein, Scheiner (2013 ▸) introduced the pnicogen bond. Meanwhile, so-called ‘π–π inter­actions’ *i.e*. the causes and effects of parallel stacking of aromatic mol­ecules, remained a disputed issue for a long time, partly due to a mistaken analogy with charge-transfer complexes (see the discussion in Hunter & Sanders, 1990 ▸) and equally fruitless explanations in terms of quadrupole moments (Williams, 1993 ▸). Each of these ‘bonds’ corresponds to a peculiar combination of the forces mentioned above, and should not be regarded as something physically unique; see the illuminating discussion by Dunitz & Gavezzotti (2005 ▸, 2012 ▸).

Somewhat aside stands a substantial family of bonds (intra- as well as inter­molecular) that can be regarded as weakened covalent bonds, or (as sometimes claimed) as ‘stills’ from the process of making/breaking a chemical bond, tracing a reaction pathway, *e.g*. of organic addition or elimination, or nucleophilic substitution (S_N_2) reactions. After enjoying high popularity in the 1980s and early 1990s (see Bürgi & Dunitz, 1994 ▸), this ‘method of mol­ecular correlations’ fell out of fashion – but probably deserves reassessment with the present-day tools.

## From geometry to energy: four steps forward and a few sideways   

Weak inter­actions are weak indeed. The total energy of a benzene mol­ecule – calculated by quantum chemistry – is 608 MJ mol^−1^. Its measured atomization energy, or sum total of covalent bond energy, is 5463±3 kJ mol^−1^, or less than 1% of the latter. The sublimation enthalpy, or sum total of inter­molecular inter­actions in a crystal, is a further two orders of magnitude lower, 43–47 kJ mol^−1^ from different measurements. Finally, the energy differences between polymorphs are usually in single units of kJ mol^−1^, comparable both to the thermal noise at room temperature (*kT* = 2.5 kJ mol^−1^) and to the error with which the sublimation enthalpy can be measured (*ca* 5 kJ mol^−1^ for organic and 24 kJ mol^−1^ for organometallic compounds, see Acree & Chickos, 2016 ▸, 2017 ▸), and beyond the reliability limits (*ca* 10 kJ mol^−1^) of the most sophisticated DFT calculations (Mackenzie *et al.*, 2017 ▸). This ranking illustrates sharply the intrinsic difficulties of analysing inter­molecular forces.

Although the theory of attractive dispersion forces (as inter­actions of instantaneous dipoles created in atoms by electrons orbiting the nuclei) was first developed by London (1930 ▸) who showed that the attractive energy is proportional to *r* ^−6^ where *r* is the inter­atomic separation; it was not until 1970 that mol­ecular mechanics calculations of lattice energy became practical. Until that time, mol­ecular crystal structures were inter­preted in terms of (i) standard van der Waals radii, compared to actual inter­molecular contact distances or used to calculate the packing density (space-filling coefficients), and (ii) hydrogen bonds and other, supposedly specific, inter­actions between individual atoms. The latter approach probably created the tradition, which persists to the present day, of generally overestimating the significance of such inter­actions, of the (often misleading) analysis of individual inter­atomic distances, and of the obsessive search for (progressively weaker) hydrogen bonds. There was, and still is, no reason to depart from the conclusion that ‘a significant share of the cohesive potential energy in organic crystals is stored in structurally non-specific mol­ecular contacts that escape a simple taxonomy’ (Gavezzotti, 2010 ▸). Furthermore, detailed analysis of inter­actions, on whatever level of sophistication, shows that the shortest (and most conspicuous) inter­molecular contacts are not stabilizing at all but repulsive, a ‘collateral damage’ of the overall optimization of mol­ecular packing (Gavezzotti, 2010 ▸).

In the period (roughly) from the 1970s to the beginning of the new millennium, supra­molecular structural chemistry was dominated by the so-called atom–atom approximation (Pertsin & Kitaigorodski, 1987 ▸; Filippini & Gavezzotti, 1993 ▸), whereby the lattice energy was represented by a sum of two-body inter­actions only, the bodies (supposedly) representing atoms in mol­ecules, with all the approximations and arbitrary conventions this entailed. Although the form of these potentials ‘descended’ from theoretical formulae, such as London’s equation, they became essentially empirical formulations, optimized to reproduce correctly macroscopic thermodynamic properties (*e.g.* sublimation enthalpy) and structural features. On these terms, atom–atom potentials worked remarkably well, but their one-to-one correspondence to physical effects was thus questionable, especially at the microscopic level.

The emergence of the Atoms in Mol­ecules (AIM) theory (Bader, 1990 ▸), which successfully rationalized intra­molecular electron density in topological terms, created false hopes of inter­molecular applications. ‘Bond paths’ were sought and found in inter­molecular space, where electron density was very low and very imprecisely determined in X-ray diffraction experiments. The fashion ended without any lasting benefit, neither in understanding nor in computational utility.

The breakthrough to a physically realistic analysis of crystal packing (made possible, of course, by the immense growth of computer capacity) started around the year 2000 with the development of Hirshfeld surface analysis (Mitchell & Spackman, 2000 ▸; Spackman & Jayatilaka, 2009 ▸), whereby the space occupied by a mol­ecule is defined by partitioning the crystal electron density into mol­ecular fragments. This greatly simplified analysing and visualizing inter­molecular contacts, in terms of topological properties of the Hirshfeld surface (shape index, curvedness) and the distances between the surface and nearest atomic nuclei, visualized as ‘fingerprint plots’ (Spackman & McKinnon, 2002 ▸). The new approach was embodied in the *CrystalExplorer* software package. At this stage, the analysis allowed is purely geometrical.

The next step was to provide a practical way to (i) calculate inter­molecular inter­actions in a sufficiently precise and physically meaningful way, and (ii) visualize the results in an informative and user-friendly manner. Several such programs have been developed (*e.g*. Johnson *et al.*, 2010 ▸). The *PIXEL* approach developed by Gavezzotti (2003*a* ▸,*b* ▸, 2010 ▸) calculates the electron density of a mol­ecule by standard quantum-chemical methods, then represents it as a large number of pixel volumes, from which the Coulombic, polarization and dispersion energies can be calculated, using atomic polarizabilities and some other, essentially empirical, approximations and adjustments, to fit experimental sublimation enthalpies. Alternatively, the new *CrystalExplorer17* software package (Mackenzie *et al.*, 2017 ▸), while using essentially the same (pixel) formalism for the Coulombic term, calculates all other energies by theoretically rigorous quantum-chemical formalisms, with adjustments to a large set of pairwise inter­action energies by high-precision quantum methods. For a given pair of mol­ecules, electrostatic, dispersion and total energy of inter­actions can be calculated and visualized separately. It is no exaggeration that the new techniques are able to revolutionize our understanding of mol­ecular crystallography.

Six of the seven papers of this issue neatly illustrate these different levels of structure inter­pretation. Yamada *et al.* (2018 ▸) report the structure of a salt with a calixarene anion, a versatile building block of inclusion compounds because of its flexible cavity. Blignaut & Lemmerer (2018 ▸) have studied a series of seven salts composed of primary amine cations and aromatic carboxyl­ate anions. In both works, the dominant supra­molecular features are strong ‘classical’ hydrogen bonds: charge-assisted N—H⋯O in the latter work, O—H⋯O in the former; the calixarene–methanol inclusion also results in O—H⋯π bonding. Correspondingly, the discussion is focused on the description of these bonds (Blignaut & Lemmerer using a graphical representation) and other short atom–atom contacts.

Canossa *et al.* (2018 ▸) compare two salts of the methyl­ene blue cation with different anions: Cl^−^ or HSO_4_^−^. In both structures the cations are similarly stacked, but in the former they form N⋯H—O bonds with water of crystallization, but in the latter have none. Surprisingly, this results in drastically different inter­planar separations *within* the stacks: 3.33 *versus* 3.55 Å, respectively, a fine illustration of the need for holistic understanding of a crystal structure. It is tantalizing, however, that the *CrystalExplorer* energy-calculating facility was not used here, although the Hirshfeld fingerpint plots were generated, but with an older version. Is the rarefied stack really destabilized in terms of energy (as the authors suggest) or is the geometry deceptive? (see above).

The Hirshfeld analysis of a CoCl_2_ complex with imidazolo­pyridine (Seth, 2018 ▸) captured a remarkable fact, not discussed in the paper: Cl⋯H contacts contribute 30% (!) of the Hirshfeld surface, although the mol­ecule contains only two chlorine atoms out of 20 potentially accessible non-hydrogen atoms – indicating the importance of electrostatic energy in this structure.

Finally, Geiger *et al.* (2018 ▸) applied the most up-to-date *CrystalExplorer17* to analyse the inter­molecular energies in the structure of HOC_6_H_4_C_6_H_4_O(CH_2_)_9_CO_2_Me, a long-chain potential gelator, while Dey *et al.* (2018 ▸) do the same for an organocatalyst PhC(O)CF_3_, a liquid under ambient conditions, which they successfully crystallized at 200 K and characterized at 110 K. It is noteworthy that in the former structure, the largest (!) energy contribution comes from C—H⋯π inter­actions between mol­ecules lying alongside each other, rather than from ‘strong’ O—H⋯O hydrogen bonds linking them head-to-tail. Concerning PhC(O)CF_3_, the most striking claim is that the stabilizing energy (−12.7 kJ mol^−1^) of mol­ecular pair III (Fig. 2) can be attributed principally, or even exclusively, to F⋯O and F⋯F *attraction*. Whereas Cl, Br and I all do form ‘halogen bonds’ with electron-donor atoms including O (see *Section 2*), F is different, having a much smaller σ-hole. Recently, Sirohiwal *et al.* (2017 ▸) suggested the existence of F⋯O halogen bonds in two other fluoro­organic compounds, based on exceptionally short F⋯O contacts in the crystal (2.71 Å, much shorter than here) and theoretical charge-density calculations; their conclusions were disputed by Jelsch & Guillot (2017 ▸), whereas an earlier experimental charge-density study by Pavan *et al.* (2013 ▸) also gave evidence of a σ-hole on fluorine and donor–acceptor F⋯F contacts.

## Non-standard conditions   

It is the *free* energy that is chemically relevant, hence the entropy effects in mol­ecular crystals should not be neglected – and at present our knowledge of these is woefully sparse. X-ray structures are seldom studied below 100 K, and for comparison with *ab initio* calculations the results must be extrapolated to 0 K. Thermal vibrations of mol­ecules in a crystal are dependent on inter­molecular forces, as the vibrating particle must ‘climb’ up the repulsive slope of the potential curve. (It is well-proven that in mol­ecular crystals, intra­molecular vibrations are insignificant compared with those of the mol­ecule as a whole.) Badenhoop & Weinhold (1997 ▸) suggested defining the ‘natural’ van der Waals contact as the distance at which the steric repulsion becomes comparable to the ambient thermal energy *kT* (2.5 kJ mol^−1^), hence the van der Waals radius must be temperature dependent. This line of research was not developed, but it is obvious that variable-temperature structural studies of mol­ecular crystals can be a substantial help in mapping the inter­molecular inter­actions.

Much more illuminating, however, can be high-pressure studies of such crystals. Indeed, thermal expansion coefficients of organic crystals being of the order of 10 ^−4^ K^−1^ (Hofmann, 2002 ▸), the volume variation over *all* practically available temperature ranges can be only a few per cent, whereas the highest compression of a mol­ecular crystal (solid H_2_) achieved so far, reduced its volume 15 times (Batsanov, 2018 ▸). Thus, Sikka (2007 ▸) analysed the correlations of O—H and H⋯O distances in O—H⋯O hydrogen bonds under pressure, observing the same correlations as had been found at ambient conditions on various compounds – *i.e*. chemically and pressure-induced deformations are similar. It was also found that the double-well potential of a hydrogen bond is transformed into a single well under pressure. A neutron diffraction study of deuterated α-glycine up to 8.7 GPa (Shinozaki *et al.*, 2018 ▸) showed non-uniform changes of hydrogen bonds on compression, with a large shrinking of bifurcated N—D⋯O and C—D⋯O bonds, while strong linear N—D⋯O bonds change little and in both senses, but in any case, Hirshfeld analysis revealed that the compression was due mainly to squeezing out of voids rather than shrinkage of these bonds. Fanetti *et al.* (2018 ▸) analysed the role of (classical) hydrogen bonds under pressure, in favouring or hindering solid-state reactions (*e.g*. polymerization of aniline). In this case, shortening *D*⋯*A* distances *do* mean stronger bonding, proven by Raman and IR spectra but, surprisingly, instead of lowering the activation energy, it stabilizes the system. This area is poorly understood and requires further research.

High-pressure diffraction experiments using diamond anvil cells (DAC) are still far from routine, notwithstanding the remarkable recent progress in design and availability of such devices (Soignard & McMillan, 2004 ▸). The working volume of a DAC, and hence of the crystal sample, is tiny and the diffraction data correspondingly weak (especially for organic crystals) and is further weakened by the absorption in the much larger diamond crystals, and often overlaps with X-ray scattering from the latter, the gasket, the ruby calibrant and the hydro­static medium. Metallic parts of a DAC also obscure a large part of the reciprocal space and make it difficult to achieve the necessary completeness of the data. All these factors are essentially unavoidable (although new DACs have wider access angles, see Moggach *et al.*, 2008 ▸), but their effects can be greatly reduced by using (i) new X-ray sources with sharper focus and higher intensity, (ii) more sensitive area detectors, especially *PILATUS* detectors, which do not accumulate noise, as well as (iii) better software, especially for absorption correction. The paper by Zakharov *et al.* (2018 ▸) in this issue, reports a comparative test, whereby the same crystal structure of the thermosalient material 1,2,4,5-tetra­bromo­benzene was determined using a state-of-the-art instrument or an older-generation diffractometer. The improvement was qualitative!

## From energy to properties   

On the whole, this field of research is still in an early stage of development. Probably the most thoroughgoing work by Pulido *et al.* (2017 ▸), is centred on developing ‘energy–structure–function’ (ESF) maps, *i.e.* combining computational crystal structure prediction and prediction of properties from the structure, the aim being to engineer highly porous crystal structures (built with intrinsically non-porous mol­ecules) for the purposes of gas storage and guest-mol­ecule selectivity. The lattice energy was calculated using anisotropic atom–atom potentials, and various tools of crystal structure prediction (CSP) were employed, including statistical analysis of known crystal structures. Verifying the ESF map predictions, a new (solvated) form of benzimidazolone was obtained, which was desolvated to yield a material with one-dimensional pores and an extremely low density (0.412 g cm^−3^).

Among the properties most directly related to the anisotropy of inter­molecular inter­actions are the anisotropies of thermal expansion, compressibility and Young’s modulus. The calculations of these properties (particularly on various polymorphs of glycine) and their experimental substanti­ation have been recently surveyed in Mackenzie *et al.* (2017 ▸).

The new capacity to calculate inter­molecular inter­actions reliably and reasonably fast opens up the fascinating prospect of reassessing some long-standing puzzles of structural chemistry. On the other hand, better algorithms for van der Waals forces, thereby developed, may prove useful for understanding and calculating two-dimensional ‘vdW-bonded’ layered materials, and mixed-dimensional vdW heterostructures (Jariwala *et al.*, 2017 ▸).

## Footnotes

1 45 years ago, I heard in a lecture by Professor A. I. Kitaigorodsky, indisputably *the* founder of organic crystal chemistry, that ‘crystal chemistry is to chemistry what autopsy is to surgery’. Great scientists sometimes also make great misjudgements.

## References

[bb1] Acree, W. & Chickos, J. S. (2016). *J. Phys. Chem. Ref. Data*, **45**, 033101.

[bb2] Acree, W. & Chickos, J. S. (2017). *J. Phys. Chem. Ref. Data*, **46**, 013104.

[bb3] Badenhoop, J. K. & Weinhold, F. (1997). *J. Chem. Phys.* **107**, 5422–5432.

[bb4] Bader, R. F. W. (1990). *Atoms in Molecules: A Quantum Theory*. Oxford University Press.

[bb5] Batsanov, S. S. (2018). *Shock and Materials.* Singapore: Springer Nature.

[bb6] Bernstein, J. (2013). *Cryst. Growth Des.* **13**, 961–964.

[bb7] Blignaut, J. & Lemmerer, A. (2018). *Acta Cryst.* E**74**, 580–586. 10.1107/S2056989017017856 PMC5947467 29850072

[bb8] Bürgi, H.-B. & Dunitz, J. D. (1994). Editors. *Structure Correlations*, Vols. 1–2. Weinheim: VCH.

[bb9] Canossa, S., Predieri, G. & Graiff, C. (2018). *Acta Cryst.* E**74**, 587–593. 10.1107/S2056989017017881 PMC5947468 29850073

[bb10] Dey, D., Sirohiwal, A. & Chopra, D. (2018). *Acta Cryst.* E**74**, 607–612. 10.1107/S2056989017016590 PMC5947471 29850076

[bb11] Dunitz, J. D. & Gavezzotti, A. (2005). *Angew. Chem. Int. Ed.* **44**, 1766–1787. 10.1002/anie.200460157 15685679

[bb12] Dunitz, J. D. & Gavezzotti, A. (2012). *CrystEngComm* **12**, 5873–5877.

[bb13] Fanetti, S., Citroni, M., Dziubek, K., Nobrega, M. M. & Bini, R. (2018). *J. Phys. Condens. Matter*, **30**, 094001. 10.1088/1361-648X/aaa8cf 29345624

[bb14] Filippini, G. & Gavezzotti, A. (1993). *Acta Cryst.* B**49**, 868–880.

[bb15] Gavezzotti, A. (2003*a*). *CrystEngComm*, **5**, 429–438.

[bb16] Gavezzotti, A. (2003*b*). *CrystEngComm*, **5**, 439–446.

[bb17] Gavezzotti, A. (2010). *Acta Cryst.* B**66**, 396–406. 10.1107/S0108768110008074 20484811

[bb18] Geiger, D. K., Geiger, H. C. & Morell, D. L. (2018). *Acta Cryst.* E**74**, 594–599. 10.1107/S2056989017016589 PMC5947469 29850074

[bb19] Hofmann, D. W. M. (2002). *Acta Cryst* B**58**, 489–493. 10.1107/s0108768101021814 12037338

[bb20] Hunter, C. A. & Sanders, K. M. (1990). *J. Am. Chem. Soc.* **112**, 5525–5534.

[bb21] Jariwala, D., Marks, T. J. & Hersam, M. (2017). *Nat. Mater.* **16**, 170–181. 10.1038/nmat4703 27479211

[bb22] Jelsch, C. & Guillot, B. (2017). *Acta Cryst* B**73**, 136–137. 10.1107/S205252061700467X 28362274

[bb23] Johnson, E. R., Keinan, S., Mori-Sánchez, P., Contreras-García, J., Cohen, A. J. & Yang, W. (2010). *J. Am. Chem. Soc.* **132**, 6498–6506. 10.1021/ja100936w PMC2864795 20394428

[bb24] London, F. (1930). *Z. Phys.* **63**, 245–279.

[bb25] Mackenzie, C. F., Spackman, P. R., Jayatilaka, D. & Spackman, M. A. (2017). *IUCrJ*, **4**, 575–587. 10.1107/S205225251700848X PMC5600021 28932404

[bb26] Metrangolo, P. & Resnati, G. (2001). *Chem. Eur. J.* **7**, 2511–2519. 10.1002/1521-3765(20010618)7:12<2511::aid-chem25110>3.0.co;2-t 11465442

[bb45] Metrangolo, P., Neukirch, H., Pilati, T. & Resnati, G. (2005). *Acc. Chem. Res.* **38**, 386–395. 10.1021/ar0400995 15895976

[bb46] Metrangolo, P., Pilati, T. & Resnati, G. (2006). *CrystEngComm*, **8**, 946–947.

[bb27] Mitchell, A. S. & Spackman, M. A. (2000). *J. Comput. Chem.* **21**, 933–942.

[bb28] Moggach, S. A., Allan, D. R., Parsons, S. & Warren, J. E. (2008). *J. Appl. Cryst.* **41**, 249–251.

[bb29] Moore, T. S. & Winmill, T. F. (1912). *J. Chem. Soc. Trans.* **101**, 1635–1676.

[bb30] Pavan, M. S., Durga Prasad, K. & Guru Row, T. N. (2013). *Chem. Commun.* **49**, 7558–7560. 10.1039/c3cc43513j 23872810

[bb31] Pertsin, A. J. & Kitaigorodski, A. I. (1987). *The Atom–Atom Potential Method.* Berlin: Springer-Verlag.

[bb32] Politzer, P., Lane, P., Concha, M. C., Ma, Y. & Murray, J. S. (2007). *J. Mol. Model.* **13**, 305–311. 10.1007/s00894-006-0154-7 17013631

[bb33] Pulido, A., Chen, L., Kaczorowski, T., Holden, D., Little, M. A., Chong, S. Y., Slater, B. J., McMahon, D. P., Bonillo, B., Stackhouse, C. J., Stephenson, A., Kane, C. M., Clowes, R., Hasell, T., Cooper, A. I. & Day, G. M. (2017). *Nature*, **543**, 657–664. 10.1038/nature21419 PMC5458805 28329756

[bb34] Scheiner, S. (2013). *Acc. Chem. Res.* **46**, 280–288. 10.1021/ar3001316 23135342

[bb35] Seth, S. K. (2018). *Acta Cryst.* E**74**, 600–606. 10.1107/S2056989018003857 PMC5947470 29850075

[bb36] Shinozaki, A., Komatsu, K., Kagi, H., Fujimoto, C., Machida, S., Sano-Furukawa, A. & Hattori, T. (2018). *J. Chem. Phys.* **148**, 044507. 10.1063/1.5009980 29390805

[bb37] Sikka, S. K. (2007). *High. Press. Res.* **27**, 313–319.

[bb38] Sirohiwal, A., Hathwar, V. R., Dey, D., Regunathan, R. & Chopra, D. (2017). *Acta Cryst.* B**73**, 140–152. 10.1107/S2052520616017492 28362276

[bb39] Soignard, E. & McMillan, P. F. (2004). Editors. *An Introduction to Diamond Anvil Cells and Loading Techniques* In *High-Pressure Crystallography*, NATO Science Series II – *Mathematics Physics and Chemistry*, Vol. 140, edited by A. Katrusiak & P. F. McMillan, pp. 81–100. Dordrecht: Kluwer.

[bb40] Spackman, M. A. & Jayatilaka, D. (2009). *CrystEngComm*, **11**, 19–32.

[bb41] Spackman, M. A. & McKinnon, J. J. (2002). *CrystEngComm*, **4**, 378–392.

[bb42] Williams, J. H. (1993). *Acc. Chem. Res.* **26**, 593–598.

[bb43] Yamada, M., Gandhi, M. R., Akimoto, K. & Hamada, F. (2018). *Acta Cryst* E**74**, 575–579. 10.1107/S2056989018001834 PMC5947466 29850071

[bb44] Zakharov, B. A., Gal, Z., Cruickshank, D. & Boldyreva, E. V. (2018). *Acta Cryst.* E**74**, 613–619. 10.1107/S205698901800470X PMC5947472 29850077

